# Oral Rehabilitation and Multidisciplinary Team Approach in Older Adult: A Narrative Review

**DOI:** 10.3390/nu18030410

**Published:** 2026-01-26

**Authors:** Mineka Yoshikawa, Azusa Haruta, Yutaro Takahashi, Shion Maruyama, Kazuhiro Tsuga

**Affiliations:** Department of Advanced Prosthodontics, Graduate School of Biomedical and Health Sciences, Hiroshima University, Hiroshima City 734-8553, Japan; harutazusa@hiroshima-u.ac.jp (A.H.); yutalo03@hiroshima-u.ac.jp (Y.T.); aster@hiroshima-u.ac.jp (S.M.); tsuga@hiroshima-u.ac.jp (K.T.)

**Keywords:** oral rehabilitation, multidisciplinary team approach, interprofessional collaboration, the older adults

## Abstract

**Background/Objectives**: Oral frailty and hypofunction in older adults are strongly associated with declines in nutritional status, physical function, swallowing ability, and overall health. Isolated interventions usually fail to achieve sufficient improvement since these conditions result from interrelated biological, psychological, and social factors. Multidisciplinary approaches combining oral management, nutritional support, and physical rehabilitation have shown promise. This narrative review synthesized evidence from 15 studies examining multifaceted interprofessional interventions across hospitals, communities, long-term care facilities, and home-care settings. **Methods**: A structured search of PubMed and Web of Science (2000–2025) identified original studies assessing oral, nutritional, or physical outcomes in older adults post-interprofessional interventions. Fifteen eligible studies were extracted; the findings were integrated using narrative synthesis owing to design and outcome heterogeneity. **Results**: Educational multidisciplinary interventions improved oral hygiene, caregiver awareness, and oral motor function. Multidisciplinary rehabilitation and multidomain programs consistently improved tongue pressure, swallowing function, mastication ability, appetite, body composition, activities of daily living, and oral intake resumption. Nutrition support team-delivered interventions reduced aspiration risks and improved oral environment and swallowing function. Community-based programs using munchy meals and combined exercises enhanced oral and physical functions. Social participation provided psychological benefits. Home-care dysphagia rehabilitation enabled 69% of tube-fed patients to resume oral intake. **Conclusions**: This narrative review supports a triadic, interprofessional approach in geriatric care, highlighting consistent improvements in oral function through integrated oral, nutritional, and rehabilitative interventions.

## 1. Introduction

Oral frailty [1] and hypofunction [2] in older adults are closely associated with frailty [3], sarcopenia [4,5], dysphagia, malnutrition, and worsening life prognosis. Oral function (OF) comprises several elements, including mastication, swallowing, speech, saliva secretion, and oral hygiene (OH), all of which are strongly correlated with the “function of eating.” In older adults, systemic disease, declining physical function, cognitive decline, and psychological and social factors, in addition to aging, combine to create a vicious cycle linking OF, nutritional status, and physical function. Therefore, assessing and addressing these factors individually is challenging, highlighting the need for a comprehensive approach. Efforts are currently being made in Japan to popularize keywords such as oral hypofunction [2] and oral frailty [1] to inform the public about the adverse effects of declining OF on the entire body, and to promote its prevention.

For mastication and swallowing disorders, medical and nursing professionals in various care settings have collaborated to implement interprofessional interventions aimed at maintaining and improving these functions. However, the environment surrounding older adults, target populations of interprofessional collaboration, the content of interventions, and outcomes measures vary greatly among studies, which can lead to fragmented knowledge in related fields. Study designs range widely—from randomized controlled trials to before-and-after comparisons and organizational intervention studies—necessitating careful organizational to effectively interpret the evidence.

Decline in OF is recognized as one of major public health problems linked to malnutrition, aspiration pneumonia, and the progression of frailty among older adults internationally. In the “Global Strategy and Action Plan on Oral Health (2023–2030),” the World Health Organization (WHO) has positioned maintaining and preventing OF’s decline as an important element of its health and longevity policy, calling for a comprehensive approach in each country [6]. In addition, based on guidelines from the National Institute for Health and Care Excellence (NICE), the UK promotes care for the older adults that incorporates assessments of masticatory and swallowing function, caregiver education, and collaboration with dental professionals [7]. Dealing with OF’s decline should be included as part of medical and nursing care policies that are not limited to the dental field.

There are few systematic reviews that comprehensively integrate OF, nutrition, and physical function to determine outcomes [8,9,10]. Therefore, there are not many interventions that combine OF, nutrition, and physical function, and there are almost no systematic reviews that integrate them.

Therefore, this narrative review aims to integrate findings from original studies that focused on multidisciplinary interventions targeting one or more aspects of oral function, swallowing function, nutritional status, and physical function in various care settings, including hospitals, communities, facilities, home care, and outpatient clinics. This will clarify the significance, feasibility, and mechanisms underlying the effectiveness of multidisciplinary collaboration for older adults, identify future challenges, and point to a direction for a comprehensive approach.

## 2. Materials and Methods

This narrative review was conducted to synthesize the current evidence on interprofessional approaches targeting OF, swallowing function, nutritional status, and physical function among older adults. A narrative synthesis was deemed appropriate since research in this field includes heterogeneous study designs, such as intervention and observational studies, and exhibits substantial variation in outcome measures and collaborative structures.

### 2.1. Search Strategy

A structured literature search was performed using PubMed and Web of Science. The search combined Medical Subject Headings and keywords related to interprofessional care, oral health, rehabilitation, nutrition, and aging. Specifically, the following terms were used in various combinations with Boolean operators (AND/OR): “interdisciplinary,” “multidisciplinary,” “interprofessional,” “oral hypofunction,” “oral frailty,” “mouth rehabilitation,” “oral health,” “rehabilitation,” “intervention,” “exercise therapy,” “nutritional status,” “nutrition,” “malnutrition,” “feeding behavior,” “elderly,” “aged,” “older adults,” “nursing home,” “long-term care,” “community-dwelling,” and “home care.” The search period spanned from January 2000 to October 2025, and only articles published in English were included. Ultimately, the database search resulted in 20 and 101 records from PubMed and Web of Science, respectively.

### 2.2. Study Selection

Two reviewers independently screened titles and abstracts of articles. Original research articles were eligible; however, review articles, case reports, and conference abstracts were excluded. Studies were included if they (i) involved older adults aged ≥65 years; (ii) assessed OF, swallowing function, nutritional status, and physical function as outcomes; (iii) implemented interprofessional interventions involving multiple health professionals (physicians, dentists, dental hygienists, dietitians, physical therapists, occupational therapists, nurses, care workers); (iv) were peer-reviewed original articles; (v) were conducted in hospitals, community settings, home care facilities, long-term care facilities, or outpatient clinics; and (iv) described the intervention procedures in sufficient detail.

The exclusion criteria included (i) interventions conducted by a single profession; (ii) studies not involving oral health–related components; (iii) review articles, editorials, and case reports; (iv) studies not targeting older adults; and (v) studies lacking clear interventions or outcome descriptions.

**Figure 1:**
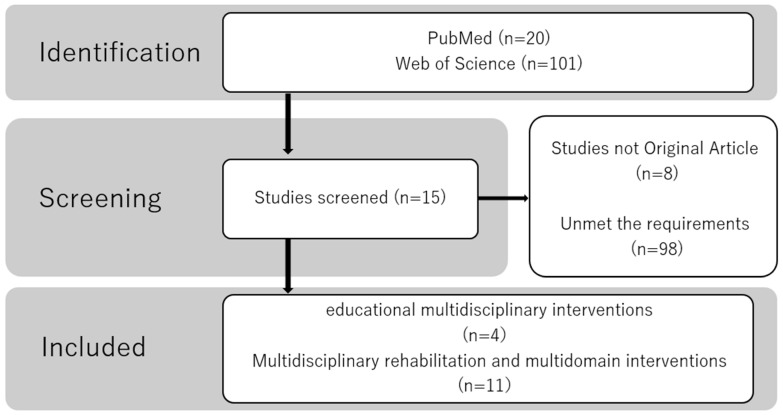
Flow diagram of the study selection process.

After full-text assessment, 15 studies with available complete PDFs and Population, Intervention, Control, and Outcome (PICO) data were available to be included in the final analysis (Figure 1).

### 2.3. Data Extraction and Synthesis

Data were extracted according to the PICO framework:

Population: Age, settings (hospital, community, home care facility, long-term care facility, outpatient clinic), and comorbidities.

Intervention: Oral health education, caregiver education, oral exercises (including tongue–lip exercises), swallowing rehabilitation, nutritional management, physical exercise, and multidomain programs.

Comparison: Usual care, physical exercise alone, and the presence/absence of a control group.

Outcomes: OF (tongue pressure, mastication, and OH), swallowing function, nutritional status, physical function, and activities of daily life (ADL).

Study design: Randomized controlled trials, pre–post studies, cohort studies, and organizational intervention studies.

Data extraction was conducted in two phases. First, information was compiled into standardized PICO tables, and missing details were supplemented through full-text review. Second, the data were thematically synthesized using narrative synthesis methods.

A quantitative synthesis (meta-analysis) was not feasible since the included studies differed substantially in terms of intervention components, collaborative structures, and outcome measures. The findings were categorized according to (i) educational multidisciplinary interventions and (ii) multidisciplinary rehabilitation and multidomain interventions, consistent with the classification used in the Results section.

Particular attention was given to commonly assessed functional domains, such as mastication, swallowing function, tongue pressure, OH, and quality of life-related indicators, to identify improvement patterns associated with interprofessional interventions as well as to describe similarities and differences across studies.

### 2.4. Risk of Bias Assessment

Risk of bias was assessed according to study design. Randomized and cluster-randomized controlled trials were evaluated using the Cochrane Risk of Bias tool (RoB 1) [11]. The methodological quality of retrospective cohort studies was assessed using the Newcastle–Ottawa Scale (NOS) [12]. Due to the lack of validated tools, before–after and case studies were not assessed for risk of bias.

## 3. Results

Fifteen studies were selected, and their characteristics are summarized in Table 1, Table 2, Table 3, Table 4 and Table 5. These studies were classified into the following two categories based on the intervention content: educational multidisciplinary interventions and multidisciplinary rehabilitation and multidomain interventions.

### 3.1. Educational Multidisciplinary Interventions

Four studies implemented interventions that involved oral health education for caregivers and care recipients.

Two studies implemented interventions using OH education alone [14,15]. Portella et al. [14] used lectures and practice conducted by dental students and a professor, along with posters on OH instruction. They observed improvements in OH status, particularly among the independent group with normal muscle strength. McNally et al. [15] conducted a 1-year educational program for direct care staff and managers and evaluated organizational changes, implementation status, and staff understanding and awareness.

Ko et al. evaluated the effectiveness of oral exercises combined with oral health education [25]. The interventions, which included OH education and tongue–lip exercises over 6 months, resulted in significant improvements in swallowing function at 6 months and in OH and tongue–lip motor function at 3 months among participants with oral hypofunction. Nutritional status and cheek bulging function improved notably after 6 months in the oral hypofunction group.

Annina et al. provided both oral health interventions and nutritional guidance for 6 months [26]. After providing OH guidance through oral hygienists and individual nutritional guidance based on dietary records, they evaluated the number of teeth with plaque and decayed teeth in the caregivers and care recipients. They clarified that the number of teeth with plaque in both participants and decayed teeth among caregivers significantly decreased.

Our findings indicate that educational multidisciplinary interventions influenced care staff awareness in the remaining study [15] and improved OH status in two studies [14,26] and enhanced OF in one [25].

### 3.2. Multidisciplinary Rehabilitation and Multidomain Interventions

Eleven studies implemented multidisciplinary interventions that included exercise and nutritional management. Six studies incorporated exercise and nutritional support in addition to oral exercise and care [13,16,17,18,19,22].

Beck et al. conducted an 11-week randomized controlled intervention study with nutrition (chocolate and homemade oral supplements), group exercise twice a week, and oral care intervention 1–2 times a week in older adult nursing home residents and compared with the normal care group [13]. After 11 weeks, the change in percentage of weight (*p* = 0.005), percentage of body mass index (*p* = 0.003), energy intake (*p* = 0.084), protein intake (*p* = 0.012), and Berg’s Balance Scale score (*p* = 0.004) was higher in the intervention group than in the control group. The percentage of participants whose functional tests improved was higher in the intervention group than in the control group. Both groups lost the same percentage of weight after the intervention (*p* = 0.908). The total percentage of weight loss from baseline to follow-up was significantly higher in the control group than in the intervention group (*p* = 0.019). Oral care was not well accepted, and the prevalence of plaque remained unchanged.

Kito et al. implemented a combined program targeting community-dwelling older individuals, consisting of gathering to eat a munchy lunch two times a week along with oral and physical exercises [16]. The control group performed only physical exercise. Notably, improvements in oral and physical function were observed. The intervention group showed a significant increase in tongue pressure, a decrease in body fat percentage and standing-up walking test time, improved walking speed, and reduced body mass index. Chewing function improved in both groups.

Matsuo et al. provided munchy lunches two times a week, oral and physical exercises, and nutritional guidance to community-dwelling older adults, while providing physical exercise alone to the control group [19]. The proportion of participants with oral dysfunction decreased from 56% to 26% (*p* = 0.002) (control group: 67% to 61%, *p* = 0.549). Furthermore, the intervention group showed significant improvements in bite force, tongue pressure, and tongue-lip movement; reduced body fat percentage; and increased muscle mass and appetite scores.

Nagano et al. [17] administered physical and occupational therapies without swallowing rehabilitation and managed appropriate nutritional intake for inpatients with orthopedic conditions at a rehabilitation hospital. These interventions resulted in significant improvements in tongue pressure, swallowing function, and ADL.

Yoshimura et al. [22] compared groups receiving oral healthcare and exercise/nutrition management alone, a combination of the two, or all rehabilitation modalities for hospitalized patients with stroke. The group receiving all rehabilitation programs showed significant improvement in ADL muscle strength and mass.

Two studies implemented interventions aimed at resuming oral intake [18,20]. Furuya et al. [18] conducted a 6-month dysphagia rehabilitation program for participants who were unable to eat orally due to dysphagia in their homes. A total of 69% of participants resumed oral intake, and maintaining the ability to walk was identified as a condition for resuming oral intake. Suzuki et al. evaluated the nutrition-intake method, swallowing ability, and oral environment at the time of referral and completion of the nutrition support term (NST) intervention in 2022 [20].

Additionally, two studies investigated the effect of an NST-mediated multidisciplinary oral health management [18,23]. In these studies, the Functional Oral Intake Scale improved, the proportion of patients unable to take oral intake decreased, the Dysphagia Severity Scale and Oral Health Assessment Tool scores significantly improved, and the incidence of aspiration decreased. They determined the impact of each oral health management method on the oral health of inpatients who received oral health management from nurses, who received instructions from dental professionals, and who were managed directly by dental professionals in 2024 [23]. Both groups showed improvements in oral health status. The denture improvement effects were higher among dental professionals than among nurses, whereas natural teeth showed only a tendency toward improvement.

Hidaka et al. [21] conducted lectures on oral health, chewing, and nutrition along with the consumption of munchy bento boxes as interventions. They successfully achieved a change in awareness, particularly among the oral frailty group.

Tuuliainen et al. provided oral self-care support and individualized nutritional counseling that led to increased dental visit rates through a heightened awareness of oral health and nutrition [24].

Hori et al. [27] conducted a 3-month OF training and nutritional guidance for patients with oral hypofunction. They reported significantly greater improvements in nutritional status and OF among these patients than among those who only received explanations of their OF test results.

Of the five studies that combined oral and physical exercises with nutritional support, four studies provided the original meal menu [13,16,19,21].

### 3.3. Risk of Bias Assessment

Randomized controlled trials were evaluated using RoB 1 [11], and every trial showed high risk of bias. As for retrospective cohort studies, one study was indicated as having high quality research using NOS [12].

## 4. Discussion

This review summarized 15 studies conducted in various settings, including hospitals, communities, nursing homes, home care facilities, and outpatient clinics, to clarify the characteristics and effects of multidisciplinary interventions primarily for masticatory and swallowing disorders in older adults. The results showed that a multifaceted approach that simultaneously addresses oral, nutritional, and physical function was consistently effective, regardless of the intervention setting or target. While each study points out the importance of integrating OF, nutrition, and rehabilitation, evidence limited to older adults requiring care and residents of nursing homes remains limited. In addition, standardization of evaluation methods, such as indicators of OF and nutrition, is also considered a future challenge.

### 4.1. Educational Approach

Four studies [14,15,25,26] that included education on OH management all showed that a multifaceted approach—combining education, behavioral change support, and daily interventions, rather than a single type of care—is effective in improving OF and OH in older adults.

The studies shared the common theme that multifaceted support tailored to the individual and environment is required. This includes OH education, establishing daily care habits, oral exercise, caregiver support, and organizational intervention. These findings suggest that simply providing OF training is insufficient to achieve adequate results. Furthermore, future studies will likely require individualized intervention designs tailored to the older individual’s level of independence and the availability of facilities and staff, since environmental factors such as physical function, caregiver burden, and the availability of organizational support affect the magnitude of intervention effects.

Furthermore, some older adults requiring care have cognitive decline, and because they have difficulty understanding instructions, there are limitations to the assessment of OF, OH management, and oral rehabilitation [28,29,30,31]. Therefore, simply providing guidance to the elderly themselves is not enough. Therefore, those around them who support them will need to make even greater efforts to address older adults’ needs, the care environment, diversity, and other factors.

### 4.2. A Trinity Approach

Five studies [13,16,17,19,22] that demonstrated a multifaceted approach showed that interventions emphasizing the interplay between OF, nutritional status, and physical function contribute to improving health outcomes in older adults. Interventions that combined OF training, nutritional support, and physical rehabilitation produced more consistent results than single-component interventions.

These studies demonstrate that OF, nutritional status, and physical function are closely interrelated, and that comprehensive, multidisciplinary interventions are more effective than approaches targeting a single domain. Furthermore, the development of more personalized programs tailored to each participant’s characteristics is required since intervention effects depend on the individuals’ level of physical independence and nutritional status.

### 4.3. From OF and Nutritional Management to Dysphagia Rehabilitation and Oral Intake Resumption

For patients receiving nutritional support in acute care hospitals (NSTs) [20,23], the following three key elements are essential for improving nutritional status and resuming oral intake: (1) improving the oral environment, (2) enhancing swallowing function, and (3) establishing an effective multidisciplinary collaborative system. Early oral care and swallowing interventions have been shown to directly improve function and restore oral intake, highlighting the importance of multidisciplinary collaboration, including medical and dental care, for the nutritional management of older adult patients. The importance of long-term feeding and swallowing interventions along with specialized nutritional approaches after oral intake resumption has also been demonstrated [18]. In addition to multidisciplinary collaborative interventions, a multidisciplinary team approach—even for the assessment of the swallowing function—has been shown to reduce aspiration pneumonia incidence in acute care wards [32]. Therefore, medical professionals should consider various approaches and share ideas.

### 4.4. Multidisciplinary Approaches in Various Medical and Nursing Care Settings

The 15 papers included in this review presented multidisciplinary approaches addressing oral, nutritional, and physical health across diverse settings for older adults.

#### 4.4.1. Multidisciplinary Approaches in Community Interventions

Multidisciplinary interventions (such as munchy lunch, oral exercises, physical exercise, and nutrition education) implemented for community-dwelling older adults contributed to improvements in tongue pressure, mastication, swallowing, oral frailty, and physical function. This intervention is gaining attention as a model for comprehensively improving oral and whole-body functions through a combination of chewing stimulation using hard ingredients, muscle strength improvement through exercise, and behavioral changes through education. Additionally, the social approach of classroom-based group participation may help prevent social frailty. For example, a correlation between low tongue pressure and depression was observed in healthy community-dwelling older adults [33]. Therefore, expanding into psychological and social approaches, such as “social connections,” “range of daily activities,” and “mentality,” is important, as these factors may precede the onset of oral frailty. Reports have also highlighted the relationship between rehabilitation efforts and the psychological aspects of nutritional improvement [34] as well as the promotion of the trinity of rehabilitation, nutrition, and OF [35]. Future approaches addressing psychological aspects will be necessary.

#### 4.4.2. The Effectiveness of Multidisciplinary Collaboration in Hospitals

Many specialists are involved in acute and convalescent rehabilitation wards. Collaboration between NSTs, dentists/dental hygienists, speech–language–hearing therapists, nurses, and rehabilitation specialists resulted in improvements in swallowing function, OH, oral intake ability, ADL, and muscle strength. This suggests that a trinity of interventions—oral management (hygiene/swallowing rehabilitation), nutritional management, and physical rehabilitation—can produce a synergistic effect in addressing complex problems such as disuse, malnutrition, and sarcopenia after acute illness. The observed association between nutritional intake (energy and protein) and improved OF supports a bidirectional relationship between nutrition and OF.

#### 4.4.3. Interventions and Collaboration in Long-Term Care Facilities

The importance of oral health in preventing the need for nursing care is well established [36,37,38]. OH education and multifaceted interventions in nursing homes generally led to greater improvements among residents with preserved physical function [14]. However, the oral care implementation rates remain an issue for older adult residents requiring assistance and those with impaired cognitive function [13]. Efforts to position oral care as a facility-wide system (e.g., through staff support systems, toolkits, standardized evaluation methods) are important for improving the quality and continuity of care. This indicates that both individuals and organizational support systems strongly influence the success of interventions.

This study found that a trinity approach addressing oral dysfunction, malnutrition, and rehabilitation was associated with favorable outcomes, and together with the high level of outcomes demonstrated in Yoshimura et al.’s study [22], this suggests that this may support the effectiveness of this integrated framework. This trinity approach addressing oral, nutritional, and physical function can be proactively implemented, particularly in medical institutions such as hospitals, where a wide range of specialists are present. However, many existing studies have limitations, such as risk of bias and heterogeneity in outcomes. Further evidence from higher-quality research is needed.

#### 4.4.4. Interventions for Older Adults Living at Home and Their Family Caregivers

Continuous swallowing rehabilitation, regular swallowing evaluations, and nutritional support from registered dietitian have increased the rate of oral intake resumption in the field of home care, as demonstrated in [18]. OH education targeted at family caregivers improved the OH of both caregivers and care recipients, highlighting the effectiveness of the novel approach of “targeting caregivers as the intervention target.” This reflects the structural characteristics of home care, in which the caregiving ability of family members directly affects the health of the individual.

#### 4.4.5. Various Oral Approaches

Within the trinity approach of oral care, nutritional management, and physical rehabilitation, those that included OF and hygiene were selected. Various approaches exist for improving OF and hygiene. However, oral approaches to older adults differ owing to differences in health insurance systems. For example, an oral care program was organized, and an OH education program for caregivers was implemented by dental students and an educator [14]. One program was also implemented by researchers affiliated with a medical school [25]. Considerable variation was also observed in cases involving dentistry. For example, in some reports [13,22,24,26], dental hygienists alone managed the oral care department, whereas in another report, a dentist led the intervention as a speaker, aiming to strengthen chewing ability through a “munchy lunch” [16,19,21]. In some cases, dentists and dental hygienists provided specialized treatment while also offering OH guidance to ward nurses, who subsequently became involved in daily oral care [20,23].

In Japan, dentists and dental hygienists provide typical specialized dental treatment, offering guidance on rehabilitation aimed at maintaining and improving OF in older adults and evaluating oral and swallowing functions [18,27]. This is considered rare worldwide. The 15 selected studies included various rehabilitation methods aimed at maintaining and improving OF, such as mouth opening exercises [39], salivary gland massage [40], and tongue coating care [41]. Tongue pressure has been reported to decrease with age [42,43,44,45,46]. Evaluation of maximum tongue pressure and guidance and management of tongue resistance training [47,48,49,50] for patients, masticatory function training using gum [51,52,53] for patients with a decline in bite force and masticatory function [54,55,56], and the intake of foods that are effective for mastication [16,18,19] are also ways for older adults to engage in oral rehabilitation while having fun. Repetitive tongue elevation and clenching have been shown to change cortical activity related to tongue muscle control to excitability [57,58]. In the context of providing OH guidance to family members and medical care staff supporting older adults requiring care [14,15], repeated learning in medical training [59], and the use of teaching materials such as videos and posters [60] are believed to contribute to various motivational and learning promotion measures.

### 4.5. Comparison with Previous Studies

Of the 15 studies included in this review, few directly evaluated nutritional status as a primary outcome, and most positioned nutrition as a fundamental element supporting the improvement of OF, swallowing, and ADL. Only a limited number of randomized controlled trials (RCTs) used Mini nutritional assessment (MNA), weight, or Body mass index (BMI) as primary endpoints, and evidence demonstrating a causal relationship between oral intervention and nutritional improvement is available. Meanwhile, there are reports that tooth loss [61], reduced masticatory function [10], and overall OF decline [9,62,63] are associated with frailty, sarcopenia, malnutrition, cognitive decline, and death, making standardization of assessment methods for oral function, nutrition, and physical function a future challenge.

The results of these 15 studies were consistent with the concept of OF decline, followed by nutritional decline, muscle weakness, frailty, and progression to the need for care [1]. They also showed high agreement with the Triad (oral care, nutritional management, and physical rehabilitation) concept reported by Yoshimura et al. [22] and Wakabayashi et al. [35]. These results are not limited to Japan but are consistent with internationally reported data in Europe, America, Asia, and South America [13,14,15,24,25,26].

Oral hypofunction disorder was officially recognized in Japan as a dental condition in 2018, and a new item for health insurance coverage was added [2]. Dental clinics across Japan can currently diagnose and manage OF decline under the health insurance system. A definition of oral frailty was established in 2024 [1], aiming to enable patients and their families to quickly assess the subtle signs of OF decline and implement appropriate measures to slow or even improve the decline. Although interventions by dental professionals are fundamental in addressing OF decline, these results also demonstrate that interventions by other medical and care professionals can further improve physical function and nutrition.

OFs are diverse, and their components are closely interrelated. The tongue plays a major role in eating, swallowing, and articulation. In studies on tongue resistance training for low tongue pressure in older adults, speech–language–hearing pathologists and dentists have conducted interventions as a single profession, observing improvements in tongue pressure [47,64,65,66]. Intervention approaches targeting multiple factors delivered by a single profession have also been reported [67]. Since the late 2010s, an increasing number of reports have been published on the relationship between OF decline and various declines in systemic function [68,69,70,71,72,73,74,75,76,77,78]. These reports show that the combination of systemic disease, decline in physical function, cognitive decline, and psychological and social factors creates a vicious cycle linking OF, nutritional status, and physical function. The need for an intervention approach addressing these factors has gradually become recognized, and recent evidence regarding the importance of an intervention approach that combines OF, nutritional status, and physical function has been recognized.

Common findings among the 15 studies presented here are as follows: (i) OF is bidirectionally related to nutritional status and physical function; (ii) enhanced nutritional management contributes to improving OF, particularly tongue pressure and swallowing; (iii) improved OH influences swallowing function and food habits; (iv) exercise supports eating behavior by improving physical function and the function of the muscles involved in swallowing; and (v) the roles of multiple professions and communication between them determine the quality of intervention.

### 4.6. Limitations

This study had some limitations. First, many studies were before-and-after comparisons, and the number of randomized controlled trials was limited. Second, the interventions were diverse and not standardized, making it challenging to compare the effects and draw causal inferences. Finally, many studies had short follow-up periods, leaving the long-term effects and sustainability of the results unclear. It should also be noted that the implementation of interventions varies by facility and region.

## 5. Conclusions

This study showed that multidisciplinary interventions integrating OF, nutritional status, and physical function are effective across hospitals, communities, and home settings. These findings support a triadic approach to preventing and improving oral frailty, nutritional decline, and functional deterioration, while emphasizing interprofessional collaboration in geriatric care.

## Figures and Tables

**Table 1:** Summary of the participant information of the selected studies (starting with the oldest).

Authors, Year Ref No.	Country	Study Design	Age	Number (Male, %)	Environment	Physical and Mental Condition	Exclusion
Beck et al., 2008 [13]	Denmark	Randomized controlled intervention study	Intervention group: 87 (mean 84–90), Control group: 86 (mean 84–87)	121 (33, 22%)	Nursing home	NA	Terminal condition, hospitalized
Portella et al., 2015 [14]	Brazil	Intervention study	60–69 (4.2%), 70–79 (36.6%), 80–89 (43.4%) ≥90 (15.8%)	120 (34, 28.3%)	Nursing home	Good cognitive status	NA
McNally et al., 2015 [15]	Canada	Case study		98 Personal care staffs	Long-term care facility residents	Personal care providers, Nurse managers, Directors of care	NA
Kito et al., 2019 [16]	Japan	A cluster randomized controlled trial	75.6 ± 5.6	86 (6, 7%)	Community-dwelling	NA	Edentulous without using dentures, food allergies, severe kidney failure, and severe dysphagia
Nagano et al., 2020 [17]	Japan	Single-arm intervention study	83.4 ± 6.5	95 (19, 20%)	Admitted for rehabilitation for orthopedic diseases/conditions	Sarcopenia	Dysphagia rehabilitation in progress, BIA contraindicated measurement, BIA measurement, error, Disability affecting tongue strength and swallowing assessment
Furuya et al., 2020 [18]	Japan	Retrospective cohort study	79.7 ± 8.9 years	116 (66, 56.9%)	Receiving home nursing care	Not eating by mouth (eternal nutrition)	Progressive neuromuscular diseases, Death or hospital admission (lost to follow-up)
Matsuo et al., 2021 [19]	Japan	A randomized controlled trial	75.6 ± 5.6	86 (6, 7%)	Community-dwelling	NA	Edentulous without dentures, Food allergy, Severe kidney failure, Severe dysphagia
Suzuki et al., 2022 [20]	Japan	Longitudinal study	71.9 ± 12.5	117 (66, 56.4%)	Consecutive inpatients	Received nutrition and oral care during NST period	<20 y, No NST nutritional care, No multidisciplinary oral care during the NST
Hidaka et al., 2023 [21]	Japan	Single-arm pre-post comparison study	72.3 ± 5.7	271 (112, 41.3%)	Community-dwelling	NA	Edentulous without dentures, Food allergy, Severe kidney failure, Severe dysphagia
Yoshimura et al., 2024 [22]	Japan	A retrospective cohort study	75.6	1012 (54.1%)	Consecutively admitted and discharged	Post-stroke	Altered consciousness at admission, Incomplete data, Consent declined
Suzuki et al., 2024 [23]	Japan	Longitudinal study	71.9 ± 12.5	117 (66, 56.4%)	Inpatients eligible for NST at an acute-care hospital	Multidisciplinary oral health management during NST	NA
Tuuliainen et al., 2024 [24]	Finland	Based on data from the population-based Nutrition, Oral Health and Medication intervention study	84.3	245 (26%)	Home care clients	(Aged 75 years or more)	NA
Ko et al., 2025 [25]	Taiwan	Intervention study	83.44 ± 10.59 (51–102)	295 (223, 75.6%)	Long-term care facility residents	NA	Mental illness, unstable vital signs, medical conditions, impaired consciousness
Annina et al., 2025 [26]	Finland	Intervention study	Caregivers: Intervention group 74.2 ± 7.7, Control group 74.6 ± 8.7, Care recipients: Intervention group 80.6 ± 6.7, Control group 79.2 ± 7.8	239 (111, 46.4%) (124 family caregivers (35, 28.2%), 115 care recipients (72, 62.6%)	Family caregivers > 60 y Dependent older adults > 65 y, Valid carer allowance	NA	Presence of end-of-life care
Hori et al., 2025 [27]	Japan	Quasi-randomized controlled trial	79 (75–85)	80 (32, 40%)	Outpatients	Oral hypofunction, periodic prosthodontic visit, training contents understood	Dysphagia, ongoing nutritional guidance, severe autoimmune xerostomia

NA, Not applicable; BIA, bioimpedance analysis; NST, Nutrition Support Team.

**Table 2:** Summary of information on rehabilitation in the selected studies (starting with the oldest).

Authors, Year Ref. No.	Professionals Involved in Interdisciplinary Team	Oral Rehabilitation	Other Rehabilitation	Rehabilitation of Control Group	Period of Intervention	Comparison
Beck et al., 2008 [13]	Physiotherapists, dental hygienists, nurses, dietitians	Oral care intervention (1–2×/week)	Nutrition, Group exercise (2×/week, 45–60 min, moderate intensity)	Normal nutrition care including oral supplements, Standard physiotherapy, Standard oral care	11 weeks	62 subjects were randomized to the intervention group
Portella et al., 2015 [14]	Dental students and a professor	Caregiver lecture (oral and body hygiene), OH instruction video Hands-on OH training (models and dentures), Image-based OH poster, Supply of toothbrushes, denture brushes and toothpaste	NA	NA	1 year	NA
McNally et al., 2015 [15]	Health professionals and researchers, Government stakeholders, Community college system, Provincial health and community association	Staff education topics and OH aids manual, Oral health promotion posters, educational videos for absent staff, mouth care toolkit selected and installed, resident-specific laminated care cards, validated nursing/OH assessment tools, oral care guiding principles and roles	NA	NA	12-month	NA
Kito et al., 2019 [16]	Principal investigator, dentists	Home oral exercises (tongue muscle strength training (Peko-panda), tongue rotation and swallowing exercise)	Physical exercises, “Munchy textured lunch” sessions, Post-exercise group meals(2×/week), Basic dietary instruction	Group physical exercise sessions for 2×/week	12 weeks	Randomly assigned into control (*n* = 43) or intervention (*n* = 43) groups
Nagano et al., 2020 [17]	Physical therapists, occupational therapists, nurses, speech therapists, dieticians		Usual physical and occupational therapy for hospitalized patients based on the Japanese health insurance system	NA	2 months	NA
Furuya et al., 2020 [18]		Dysphagia rehabilitation	NA	NA	6 months	NA
Matsuo et al., 2021 [19]	Principal investigator	COPE-TeL program (home oral exercises (tongue muscle strength training using Peko-panda^TM^), tongue rotation and swallowing exercise	COPE-TeL program (Group physical exercises, “Munchy textured lunch” session, Post- exercises group meals, Basic dietary instruction	Only the physical exercise regimen	12 weeks	Randomized to control or intervention, Subgrouped (OHF/NOF) by baseline oral examination
Suzuki et al., 2022 [20]	NST team (physicians, nurses, dentists, dental hygienists, dietitians, pharmacists, physical therapists, speech therapists, and other professionals)	Dental treatment by dentists, professional oral care by dental hygienists, trained ward nurses’ oral care, dysphagia rehabilitation by speech therapists	NA	NA	During NST intervention period	Compare nutrition-intake swallowing and oral status, At NST referral vs. post intervention
Hidaka et al., 2023 [21]	Dieticians, dental professionals	Comprehensive awareness modification of mouth	CAMCAM program (gather once a month at community centers to learn about oral health and nutrition while eating a “munchy” textured lunch containing proper nutrition)	Same	6 months	Grouped by OFI-8 (oral frailty vs. robust), Tested KCL and CAMCAM differences and score changes
Yoshimura et al., 2024 [22]	Rehabilitation therapists, dietitians, dental hygienists	Oral management by dental professionals	Intensive rehabilitation, personalized nutrition support	NA	During the inpatient recovery period	FIM-motor at discharge and its gain, and discharge scores of SMI and HGS
Suzuki et al., 2024 [23]	Nurses, dental health care professionals	Oral health management by trained nurses (Ns), oral health management by dental professionals (D)	NA	NA	until NST end	At the start and the end of the NST intervention
Tuuliainen et al., 2024 [24]	Trained home care nurses, pharmacists, nutritionists and dental hygienists	OH instructions (written and verbal), plaque and interdental cleaning guidance, Denture cleaning and storage guidance, symptoms management instructions	Individualized nutritional plan increasing meals, energy, protein, fluids	NA	6 months	Baseline and after a 6-month follow-up comparison, health and oral characteristics, oral care use in prior 12 months
Ko et al., 2025 [25]	Research staff, caregivers, nursing staffs	OH education, tongue–lip exercises, oral cleaning methods, increasing oral cleaning frequency, daily oral/bedside speech exercises	NA	NA	6 months	NA
Annina et al., 2025 [26]	Nurse, clinical nutritionist, dental hygienist	Tailored oral health intervention for family caregivers, verbal and written instructions, brushing, interdental cleaning, dentures, oral mucosa and dry mouth care	NA	Control: baseline interviews and examinations only, No caregiver intervention	6 months (oral health guidance at baseline and 2 months visits)	Randomized to intervention or control, caregivers and care recipient groups
Hori et al., 2025 [27]	Dentist	daily OF training, OH, dryness, occlusal force, tongue–lip, tongue pressure, mastication and swallowing decline	Dietary Advice	None (only examined and explained all findings)	3 months	Oral hypofunction patients divided into intervention vs. control

NA, Not applicable; OH, oral hygiene; COPE-TeL program, comprehensive oral and physical exercises and textured lunch gatherings; OHF, oral hypofunction; NOF, normal OF; NST, Nutrition Support Team; CAMCAM, Chewing And Meal; OFI-8, Oral Frailty Index-8; KCL, The self-administered Kihon Checklist; FIM, Functional Independence Measure; SMI, Skeletal muscle mass index; HGS, handgrip strength; OF, oral function.

**Table 3:** Summary of assessment items and results in the selected studies (starting with the oldest).

Authors, Year	Assessment Items (OF, Oral Health)	Assessment Items (Other)	Timing of Assessment	Main Results
Beck et al., 2008 [13]	Prevalence of plaque	MDS (height and weight, BMI, dietary intake, HGS, Senior Fitness Test, Berg’s Balance Scale)	4 months after the end of the intervention	After 11 weeks the change in percentage of weight (*p* = 0.005), percentage of BMI (*p* = 0.003), energy intake (*p* = 0.084), protein intake (*p* = 0.012), and Berg’s Balance Scale (*p* = 0.004) was higher in the intervention group than in the control group. The percentage of subjects whose functional tests improved was higher in the intervention group. Both groups lost the same percentage of weight after the intervention (*p* = 0.908). The total percentage of weight loss from baseline to follow-up was higher in the control group (*p* = 0.019).
Portella et al., 2015 [14]	MPS	Katz Index for activities of daily living, HGS	Baseline and 1 year after intervention	The MPS was significantly reduced (*p* = 0.001) at follow-up; however, a separate analysis showed that only the independent elderly (*p* = 0.002) and those with normal muscle strength (*p* = 0.006) showed a reduction in MPS during the follow-up examination.
McNally et al., 2015 [15]	Oral care activities records	Brief targeted interview, Education evaluations	12-week intervals	The oral care intervention resulted in heightened awareness, support and greater efficiency amongst the care team. The presence of a “champion” was a key feature for sustaining processes. Management had a clear role to play to ensure support and accountability for the intervention
Kito et al., 2019 [16]	Occlusal force, tongue pressure, tongue–lip motor function, masticatory function	BMI, body fat percentage, SMI, HGS, UWS, TUG	Baseline, after 12 weeks	OF as measured by tongue pressure increased significantly in the intervention group (*p* = 0.031), but not in the control group.
Nagano et al., 2020 [17]	Tongue strength, MASA	FIM, BMI, MNA-SF, SMI, HGS, the amount of energy and protein intake, intervention time	Baseline and after two months of intervention or discharge within two months	The mean tongue strength after the intervention was significantly increased from 25.4 ± 8.9 kPa to 30.5 ± 7.6 kPa as a result of the treatment (*p* < 0.001).
Furuya et al., 2020 [18]	FOIS, history of pneumonia, duration of enteral nutrition, BMI, alertness, physical function, swallowing function		The initial examination, after 6 months of rehabilitation	FOIS scores increased significantly after 6 months rather than those at the initial evaluation (*p* < 0.001).
Matsuo et al., 2021 [19]	OF (OH, oral wetness, occlusal force, tongue–lip motor function, tongue pressure, masticatory function, EAT-10)	Body composition, BMI, SMI, body fat mass percentage (BFM%), HGS, UWS, CNAQ, MNA-SF	12 weeks	Physical function, such as hand grip strength and walking speed, was significantly lower in the OHF group at the initial assessment. The proportion of participants with OHF was 56% in the intervention group and 67% in the control group before the trial, which became significantly reduced after completing the COPE-TeL program in the intervention group (26%, *p* = 0.002), but not in the controls (61%, *p* = 0.549).
Suzuki et al., 2022 [20]	FOIS, DSS, OHAT	Demographic data, CCI, JCS	At the time of referral and completion of the NST intervention	FOIS, DSS, and OHAT scores showed significant improvements (*p* < 0.001). Even after adjusting the results for systemic parameters, FOIS score improvement correlated positively with the length of NST intervention (*p* = 0.030) and DSS score improvement (*p* < 0.001) as well as OHAT score improvement (*p* = 0.047).
Hidaka et al., 2023 [21]	CAMCAM checklist, OFI-8, Chew 20	SNAQ, KCL	Before and after the 6-month CAMCAM program	KCL and CAMCAM checklist scores were significantly lower in the oral frailty group at the initial assessment. OFI-8 and KCL findings were significantly improved in the oral frailty group after completing the program (all *p* < 0.05).
Yoshimura et al., 2024 [22]	ROAG, FIM	HGS, SMI	At admission, at discharge	The combination of all three interventions demonstrated the strongest association with improved ADL.
Suzuki et al., 2024 [23]	OHAT	CCI, JCS, PS	Until the end of the NST intervention	The participants received oral health management from nurses and dental professionals showed significant improvements in the total OHAT scores at the end of the NST intervention.
Tuuliainen et al., 2024 [24]	Asked about the following factors related to need for care; subjective oral health, self-perceived need for dental care, whether the participant had experienced toothache or other oral discomfort over the past 12 months, difficulties in cleaning mouth or teeth, and/or whether they had difficulties in eating, denture status	MMSE, ADL, IADL	Within the previous year at baseline and after the 6-month follow-up	At baseline, 43% of participants reported visits to oral health care within the previous year. At 6-month follow-up, this proportion was 51%. In the intervention group, the corresponding figures were 46% and 53%, and in the controls 39% and 48%. Adjusted regression analyses showed that this change was statistically significant (*p* = 0.008).
Ko et al., 2025 [25]	Oral health, swallowing function (EAT-10, TCI, OHAT, tongue pressure, tongue–lip motor function, MNA-SF)	BMI, nutritional status, frailty (SOF, HGS, cheek bulging)	The first evaluation, after 3 months, after 6 months	The prevalence of oral hypofunction in the participants was 58.3%. The intervention led to significant improvements in swallowing function 6 months later and in OH and tongue–lip motor function 3 months later in the participants with oral hypofunction.
Annina et al., 2025 [26]	Information on brushing of teeth and dentures, Information on interdental cleaning, cleaning, and storing of dental prostheses, cleaning of oral mucosa, and dry mouth care, periodontal probing and measuring, the presence, type, and location of removable dental prostheses, denture hygiene, examination the oral mucosa, The number and condition of the teeth, modified Silness and Löe Index (the presence of plaque)	MNA, weight, height, daily eating routines, information on sociodemographic factors, medication, health status, IADL, ADL, depressive symptoms, and cognitive functioning, FCI, GDS-15, MMSE	6-month, 12- month visits	The number of teeth with plaque decreased among family caregivers (β = −2.1, CI −4.0–(−1.2), *p* = 0.015) and their care recipients (β = −0.6, CI −0.0–(−2.1), *p* = 0.050). The number of teeth with caries decreased among family caregivers who participated in the intervention (β = −0.6, CI −1.1–(−0.1), *p* = 0.015), but not among their care recipients.
Hori et al., 2025 [27]	OF tests (OH, oral dryness, occlusal force, tongue–lip motor function, tongue pressure, masticatory function, swallowing function)	BMI, DVS, MNA score, CNAQ	Baseline, after 1.5 months, after 3 months	The intervention group exhibited a significant increase in the mean MNA score over the study period (baseline: 25.4 ± 3.2; after 3 months: 26.3 ± 3.0), whereas no significant difference was observed in the control group (baseline: 26.4 ± 2.4; after 3 months: 26.4 ± 2.7).

OF, oral function; NA, Not applicable; MDS, minimum data set; BMI, body mass index; HGS, handgrip strength; MPS, mucosal plaque score; SMI, skeletal muscle mass index; UWS, usual walking speed; TUG, time of timed up and go test; MASA, Mann Assessment of Swallowing Ability; FIM, Functional Independence Measure; MNA-SF, Mini Nutritional Assessment-Short Form; FOIS, Functional Oral Intake Scale; OH, oral hygiene; BFM, body fat mass; CNAQ, Council on Nutrition Appetite Questionnaire; OHF, oral hypofunction; COPE-TeL program, comprehensive oral and physical exercises and textured lunch gatherings; DSS, Dysphagia Severity Scale; OHAT, Oral Health Assessment Tool; CCI, Charlson Comorbidity Index; JCS, Japan Coma Scale; NST, Nutrition Support Team; CAMCAM, Chewing And Meal; OFI-8, Oral Frailty Index-8; Chew 20,Chew Score 20; SNAQ, Simplified Nutrition Appetite Questionnaire; KCL, The self-administered Kihon Checklist; ROAG, The Revised Oral Assessment Guide; ADL, Activities of Daily Living; PS, Performance status; MMSE, Mini-Mental State Examination; IADL, Instrumental Activities of Daily Living; EAT-10, Eating Assessment Tool; TCI, Tongue Coating Index; SOF, The Study of Osteoporotic Fractures; FCI, Functional Comorbidity Index; GDS, 15-item geriatric depression scale; DVS, Dietary Variety Score.

**Table 4:** Summary of nutritional assessment items and results in the selected studies (starting with the oldest).

Authors, Year Ref. No.	Nutritional Assessment Index	The Role of Nutritional Assessment	Treatment of Nutritional Outcomes
Beck et al., 2008 [13]	Weight, BMI, energy/protein intake	Primary outcome	Significant improvement in nutritional status
Portella et al., 2015 [14]	None	Theoretical background	No nutritional outcomes
McNally et al., 2015 [15]	None	Theoretical background	Nutrition described conceptually only
Kito et al., 2019 [16]	Food education lecture	Assumption/background	No nutritional outcomes
Nagano et al., 2020 [17]	Energy, protein intake	Effect modifier	Nutritional intake correlates with improved tongue pressure
Furuya et al., 2020 [18]	BMI (baseline characteristic)	Background factor	Changes in nutritional status not evaluated
Matsuo et al., 2021 [19]	Food education lecture	Assumption/background	No nutritional outcomes
Suzuki et al., 2022 [20]	FOIS	Primary outcome	Improved oral intake levels
Hidaka et al., 2023 [21]	Food education lecture	Assumption/background	No nutritional outcomes
Yoshimura et al., 2024 [22]	Individual nutrition support	Intervention	Secondarily, improvements in ADL and HG
Suzuki et al. 2024 [23]	BMI (baseline characteristic)	Background factor	No nutritional outcomes
Tuuliainen et al. 2024 [24]	MNA, albumin, prealbmin, dietary survey	Indicator for intervention design	Behavioral outcomes (raising nutrition awareness)
Ko et al., 2025 [25]	Nutritional status (e.g., BMI.)	Secondary outcomes	Improvement in nutritional status
Annina et al., 2025 [26]	MNA, 3-day dietary record	Supplementary assessments	Nutritional changes reported separately
Hori et al. 2025 [27]	MNA, BMI, DVS, CNAQ	Primary outcome	

NA, Not applicable; BMI, body mass index; FOIS, Functional Oral Intake Scale; ADL, Activities of Daily Living; MNA, Mini Nutritional Assessment; DVS, Dietary Variety Score; CNAQ, Council on Nutrition Appetite Questionnaire.

**Table 5:** Bias risk in the selected studies (starting with the oldest).

Authors, Year Ref. No.	Design	RoB Tool	Selection Bias	Comparability/Allocation	Performance Bias	Detection Bias	Attrition Bias	Reporting Bias	Other Bias	Overall Judgment
Beck et al., 2008 [13]	RCT	RoB 1	High	High	High	Low	Low	Unclear	High	High
Portella et al., 2015 [14]	Before–after study	Not assessed	NA	NA	NA	NA	NA	NA	NA	NA
McNally et al., 2015 [15]	Case study	Not assessed	NA	NA	NA	NA	NA	NA	NA	NA
Kito et al., 2019 [16]	Cluster RCT	RoB 1	Low	Low	High	Low	Low	Low	Unclear	High
Nagano et al., 2020 [17]	Before–after study	Not assessed	NA	NA	NA	NA	NA	NA	NA	NA
Furuya et al., 2020 [18]	Retrospective cohort study	NOS	★★★	★	★★	NA	NA	NA	NA	6
Matsuo et al., 2021 [19]	Cluster RCT	RoB 1	Low	Low	High	Low	Low	Low	Unclear	High
Suzuki et al., 2022 [20]	Before–after study	Not assessed	NA	NA	NA	NA	NA	NA	NA	NA
Hidaka et al., 2023 [21]	Before–after study	Not assessed	NA	NA	NA	NA	NA	NA	NA	NA
Yoshimura et al., 2024 [22]	Retrospective cohort study	NOS	★★★	★★	★★★	NA	NA	NA	NA	8
Suzuki et al. 2024 [23]	Before–after study	Not assessed	NA	NA	NA	NA	NA	NA	NA	NA
Tuuliainen et al. 2024 [24]	RCT	RoB 1	High	High	High	High	High	Low	High	High
Ko et al., 2025 [25]	Before–after study	Not assessed	NA	NA	NA	NA	NA	NA	NA	NA
Annina et al., 2025 [26]	RCT	RoB 1	Unclear	High	High	High	High	Unclear	High	High
Hori et al. 2025 [27]	RCT	RoB 1	High	High	High	Unclear	Low	Low	Unclear	High

NA, Not applicable; RCT, randomized controlled trial; RoB, risk of bias; NOS, Newcastle–Ottawa quality assessment scale; High, high risk; Low, low risk. Risk-of-bias domains were conceptually harmonized to enable comparison across heterogeneous study designs and assessment tools. Randomized controlled trials and cluster randomized controlled trials were assessed using the Cochrane Risk of Bias tool (RoB 1), while retrospective cohort studies were assessed using the Newcastle–Ottawa Scale (NOS). For NOS-assessed studies, stars represent the quality score for each domain, with a higher number of stars indicating higher methodological quality. Before–after studies and case studies were not formally assessed due to the absence of validated risk-of-bias assessment tools for these study designs.

## Data Availability

Not applicable.

## Abbreviations

The following abbreviations are used in the manuscript:

WHO	World Health Organization
NICE	National Institute for Health and Care Excellence
OF	Oral function
OH	Oral hygiene
ADL	Activities of daily living.
PICO	Population, Intervention, Control, Outcome
RoB1	Cochrane risk of bias tool
NOS	Newcastle-Ottawa scale
NST	Nutrition support team
MNA	Mini nutritional assessment
BMI	Body mass index
RCT	Randomized controlled trial
